# Alimentary and Pharmaceutical Approach to Natural Antimicrobials against *Clostridioides difficile* Gastrointestinal Infection

**DOI:** 10.3390/foods10051124

**Published:** 2021-05-19

**Authors:** Miguel Tortajada-Girbés, Alejandro Rivas, Manuel Hernández, Ana González, Maria A. Ferrús, Maria C. Pina-Pérez

**Affiliations:** 1 Department of Pediatrics, University Dr. Peset Hospital, Avda, de Gaspar Aguilar, 90, 46017 Valencia, Spain; miguel.tortajada-girbes@uv.es; 2 Departmento Tecnología de Alimentos, Escuela Técnica Superior de Ingeniería Agronómica y del Medio Natural (ETSIAMN), Universitat Politècnica de València, Camino de Vera s/n, 46022 Valencia, Spain; alriso@tal.upv.es; 3 Departmento Biotecnología, Escuela Técnica Superior de Ingeniería Agronómica y del Medio Natural (ETSIAMN), Universitat Politècnica de València, Camino de Vera s/n, 46022 Valencia, Spain; mhernand@btc.upv.es (M.H.); angonpel@btc.upv.es (A.G.); mferrus@btc.upv.es (M.A.F.); 4 Departmento Microbiologia y Ecología, Facultad Ciencias Biológicas, Universitat de València, C/Dr. Moliner, 50, 46100 Burjassot, Spain

**Keywords:** *Clostridioides difficile*, marine bioactives, algae, fucoidan, natural antimicrobials, diet, microbiome, gastrointestinal infection

## Abstract

Incidence of *Clostridioides difficile* infection (CDI) has been increasing in recent decades due to different factors, namely (i) extended use of broad-spectrum antibiotics, (ii) transmission within asymptomatic and susceptible patients, and (iii) unbalanced gastrointestinal microbiome and collateral diseases that favor *C. difficile* gastrointestinal domination and toxin production. Although antibiotic therapies have resulted in successful control of CDI in the last 20 years, the development of novel strategies is urged in order to combat the capability of *C. difficile* to generate and acquire resistance to conventional treatments and its consequent proliferation. In this regard, vegetable and marine bioactives have emerged as alternative and effective molecules to fight against this concerning pathogen. The present review examines the effectiveness of natural antimicrobials from vegetable and algae origin that have been used experimentally in in vitro and in vivo settings to prevent and combat CDI. The aim of the present work is to contribute to accurately describe the prospective use of emerging antimicrobials as future nutraceuticals and preventive therapies, namely (i) as dietary supplement to prevent CDI and reduce CDI recurrence by means of microbiota modulation and (ii) administering them complementarily to other treatments requiring antibiotics to prevent *C. difficile* gut invasion and infection progression.

## 1. Introduction

Since 2017, following the first publication of the most antibiotic-resistant bacteria by the World Health Organization (WHO) [1], there has been an urgent call internationally to boost research and development on novel strategies based on natural or synthetic antibiotics to effectively fight against these microorganisms. In fact, it is expected that by 2050, more people will die due to multiresistant bacteria than cancer pathologies [1]. Among these highly antimicrobial-resistant bacteria, *Clostridioides difficile* is becoming a concerning threat worldwide [2,3,4]. These Gram-positive anaerobic and spore-forming bacteria has become the most frequent causal agent of hospital-acquired intestinal infection in Europe and all over the world, causing close to 30,000 death per year in the US (estimated mortality close to 16.7%) [3,5]. According to a recent surveillance (2019) developed by the European Centre for Disease Prevention and Control (ECDC) in collaboration with the US Centers for Disease Control and Prevention (CDC), the incidence of *C. difficile* infections (CDI) has increased close to 70% in relation to values included in the previous European surveillance study (2012) [6]. The consequences of CDI are fatal in some cases, with high rates of morbidity and mortality (16–23%) [5], starting with diarrhea, which can result in major complications including loss of intestinal barrier function, pseudomembranous colitis, toxic megacolon, colon perforations, and sepsis [2,7]. CDI is caused by the bacterial production of two toxins, A and B, and also a third binary toxin produced by some strains of *C. difficile* (including the hypervirulent NAP1/027 epidemic strain) [8]. High rates of CDI recurrence have also been progressively detected in primary infected patients managed under an antibiotic treatment, with close to 20–25% of infected and recovered patients suffering a second episode [9].

The bacterium *C. difficile* is spread via the fecal–oral route. The progression of colonization and infection occurs via two routes, namely the presence (endogenous infection) or acquisition (exogenous infection) of CD and the altered composition of gastrointestinal microbiota. Several factors have been described as being responsible for the increased incidence of CDI and its fatal consequences in recent years. Among them, one of the most significant reasons for CD microbiota domination and severe gut infection is linked to the exposure of patients to broad-spectrum antibiotics against which CD is resistant, thereby favoring extensive gastrointestinal colonization and toxin production [9,10]. Some of the antibiotics able to disrupt the healthy microbiota balance in the gastrointestinal tract (GIT), thereby allowing proliferation of *C. difficile,* are ampicillin, amoxicillin, cephalosporins, clindamycin, and fluoroquinolones [9].

Other relevant factors in relation to CDI progression and the severity of its consequences include (i) age, (ii) disruption of the host defense (low serum antibody response to *C. difficile* toxins), and (iii) previous health status of patients [9,10,11]. Important research efforts are nowadays focused on further understanding the observed increase in CDI infection within communities rather than just in specific healthcare or hospital facilities [6]. Asymptomatic patients act as agents of pathogen reservoirs and vehicles of infection transmission to immune-compromised individuals [9,10,11]. 

### Current Antibiotic Therapies Applied against Clostridioides difficile

Among the most effective antibiotics used to date against CDI are vancomycin, fidaxomicin, and metronidazole, which have been applied as the first line of therapy in the last 30 years. Antibiotics targeted at inactivating *C. difficile* act mainly against bacterial DNA (by means of DNA damage), causing inhibition of protein synthesis and enzymatic activity (pyruvate and ferredoxin oxidoreductase) or disruption of the membrane potential and peptidoglycan synthesis [2,12,13]. A significant reduction in the effectiveness of antibiotic therapies has been observed in recent years. This includes increased resistance and “resistome” transference in *C. difficile* as well as the emergence of new hypervirulent strains [12]. According to Peng et al. [2], in recent years, causative events resulting in increased *C. difficle* antibiotic resistance include (i) transfer of mobile genetic elements, (ii) selective pressure in vivo resulting in gene mutations, (iii) altered expression of redox-active proteins, (iv) iron metabolism, (v) DNA repair, and (vi) biofilm formation [2]. Failure rates with authorized treatments are in the range of 14–22% (14% with vancomycin and 22% with metronidazole), while the recurrence rate is also high (25–30%) [3]. Similar results have been observed for the most recently applied treatment with fidaxomicin, with 12–15% infection recurrence observed in recent decades (2009–2021).

In the last 20 years, very few new antibiotics have been successfully developed against CDI. Among the most recently developed antibiotics are cadazolid, surotomycin, ramoplanin, nitazoxanide, rifampin, and rifaximin [14,15]. Unique properties to fight against *C. difficile* have been attributed to cadazolid (minimum inhibitory concentration (MIC) = 0.125 µg/mL; minimum bactericidal concentration (MBC) (3 log_10_ reduction) = 2 × MIC). The mode of action of this antibiotic is focused on protein synthesis inhibition (toxin inhibition) and suppression of spore formation, which increases *C. difficile* susceptibility to treatment. Among the main advantages of this compound are (i) strong in vitro and in vivo effectiveness; (ii) the capability to inhibit *C. difficile* infection in a gut model, thereby maintaining normal microbiota at correct levels; and (iii) reduced rates of infection recurrence [15]. Other novel drugs are nowadays in Phase 1, 2, and 3 trials, with ibezapolstat (ACX-362E), CRS3123, and NVB302 the most recently developed drugs for the oral treatment of CDI [3]. 

However, under pressure, the *C. difficile* genome sets up a variety of resistance mechanisms responsible for the observed capability of CD to persist and be recurrent even when clinic antimicrobial strategies are applied. In fact, conjugation, transduction, and/or transformation of mobile genetic entities, and specifically transposons, within different *C. difficile* strains and/or between *C. difficile* and other bacterial species are among the remarkable resistance mechanisms associated with this microorganism. Additionally, acquired antibiotic resistance by means of alterations in antibiotic targets and/or metabolic pathways has been described as significant contributing factors to proliferation and increased incidence of CDI, particularly in the last decade. In fact, aggressive symptomatology hypervirulent strains have been emerging in recent times (2010–2020), including *C. difficile* BI/NAP1/ribotype 027 and BK/NAP7/ribotype 078, which are resistant to fluoroquinolones and cephalosporins. This has contributed to the increase in antibiotic resistance, along with other relevant exacerbating virulence factors such as increased sporulation and surface layer protein adherence capability of these strains [8].

The biofilm-forming capacity of *C. difficile* has significantly contributed to the increase in antibiotic resistance [16,17]. In fact, according to Semenyuk et al. [18], *C. difficile* biofilms confer a 100-fold increase in metronidazole resistance [18]. It has been proven that the capacity of sessile bacteria to form biofilms in the mucus layer of the gut plays a fundamental role in gut health and disease. Although very little information has been published to date regarding the in vivo biofilm-forming capacity of *C. difficile*, it is well known that these multicellular structures could potentially protect bacteria from cellular immune responses and from antibiotics [16,17]. Moreover, the recurrence of CDI can be associated with biofilm persistence [17]. At present, among the most concerning unknown aspects of clostridial pathogenesis (gut colonization and infection progression) is the biofilm-forming capacity of *C. difficile* in vivo and how this multicellular intraspecific “dialogue” can interact with the host immune system [17].

In spite of the urgent need to develop novel antimicrobial therapies against this pathogen and the recent technological advancements in vaccination, the process of research, development, validation, authorization, and launch of any novel drug represents an average cost of USD 2–3 billion and takes up to 13–15 years [19]. A very common approach to find new antimicrobial options is the study of currently authorized drugs, even those applied in other clinical areas (e.g., oncology, dermatology, and digestive medicine), as well as the search for synergies between effective antibiotics that are currently used. In this regard, Pal and Seelem [19] reported some of the natural oncological drugs that show potent anticlostridial effect, including mitomycin C (MIC = 0.5μM), plicamycin/mithramycin A (MIC ≤ 0.25 μM), aureomycin (MIC = 0.5μM), siomycin A (MIC ≤ 0.25 μM), tetrocarcin A (MIC = 0.5 μM), rifamycin (MIC ≤ 0.25 μM), nigericin (MIC ≤ 0.25 μM), antibiotic X-536A (MIC = 1 μM), chaetochromin (MIC = 0.5 μM), and levomycin (MIC ≤ 0.25 μM). The chemotherapeutic mitomycin C has previously shown antibacterial activity against planktonic, biofilm, and metabolically dormant persister cells of *E. coli*, *Staphylococcus aureus*, and *Pseudomonas aeruginosa* (MIC within 0.2–15 μg/mL). The anti-CD mitomycin effect is exerted with a MIC value of 0.25 μg/mL. Naclerio et al. [20] recently developed one of the most effective antimicrobials against CD, the trifluoromethylthio-containing N-(1,3,4-oxadiazol-2-yl)benzamides, which displayed very potent activity with MIC values as low as 0.003 (µg/mL) [20]. According to the study by Naclerio et al. [20], this compound (which is nontoxic to mammalian cells) can be obtained by the replacement of the thiophene toxicophore molecule in TFOB (named as compound 12 by Naclerio et al.) to generate the HSGN-218 product. The principal antimicrobial potential of this compound is mainly attributed to the (trifluoromethylthio)phenyl group, which shows even more effectiveness than vancomycin against *C. difficile* (MIC values ranging between 0.25 and 1 μg/mL) [20]. Another chemically potent compound, 2-(4-(3-(trifluoromethoxy)phenoxy)picolinamido)benzo[d]oxazole-5-carboxylate, with high selectivity against *C. difficile* was discovered by Speri et al. [21]. The selectivity of this compound to exclusively target *C. difficile* was indicated by an MIC value of 0.125 µg/mL compared to MIC values against beneficial microbiota (*Bifidobacterium fragilis*, *Lactobacillus reuteri*, and *Bifidobacterium longum*) of 2–126 µg/mL.

In the search for alternative natural antimicrobial strategies, other molecules have demonstrated bactericidal or bacteriostatic effect against *C. difficile.* To date, these emerging studies to test the antimicrobial potential of different natural antimicrobial compounds against *C. difficile* have mainly been based on the in vitro disk diffusion test methodology followed by comparison with the Clinical and Laboratory Standards Institute (CLSI) breakpoint methodology that is applied for conventionally used antibiotics [2,13,15], with some studies validating their findings by means of in vivo animal models [9,22]. 

**Figure 1:**
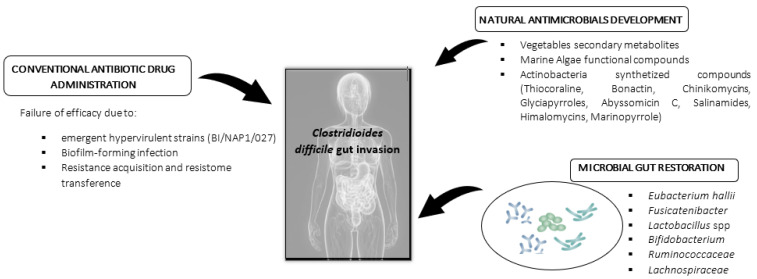
Conventional and novel strategies applied in *Clostridioides difficile* gastrointestinal infection treatment.

The present review aims to provide a global view on the most effective alternative antimicrobials found in vegetable, bacterial, and marine sources against *C. difficile* and the possibilities of these materials to exert inhibitory and bactericidal potential in order to contribute to increasing the current knowledge on future clinic and nutraceutical supplements to be administered as therapy in CDI mitigation (Figure 1). In the present study, a review was performed based on published literature on PubMed, Google Scholar, EMBASE, BIOSIS, and Web of Science databases from 2000 to 2021. The terms included to obtain results were as follows: “natural antimicrobials”, “marine bioactives”, “*Clostridium difficile*”, “therapy”, “marine antimicrobials”, “marine drugs”, “algae”, and “gastrointestinal disease”. 

## 2. Natural Antimicrobials against *Clostridioides difficile* Infection (CDI): Nutraceutical and Pharmaceutical Approach

Nowadays, in addition to previously detailed antibiotic therapies authorized and generally used in CDI treatment, novel materials and bioactives are being investigated (Table 1). Among the most innovative novel substances with possible application in nutraceutical CDI management are (i) natural compounds from vegetable origin, (ii) restoration of beneficial microbiota, and (iii) marine (bacterial and algae) bioactive compounds. 

### 2.1. Vegetable Compounds in Clostridioides difficile Infection Mitigation: Research Advances

Natural antimicrobials extracted from vegetable materials (fruits, seeds, grains, leaves, roots, and vegetables) or by-products are representing an innovative and sustainable pathway to fight against human clinical and foodborne pathogens [36,37,38]. Effectively, natural vegetable raw materials (and extracted/processed products) have demonstrated antimicrobial potential against *C. difficile*. Recently, Roshan et al. [24] assayed the in vitro antimicrobial potential of natural onion and garlic juices (100% *v/v*), onion and garlic powders (20% *w/v*), ginger, artichoke, honey, cinnamon powder (20% *w/v*), turmeric powder (20% *w/v*), and aloe vera compounds against different pathogenic strains of *C. difficile* (via disk diffusion method and microdilution test). Results revealed that, among the assayed products, garlic juice (100% *v/v*) was the most effective in inhibiting *C. difficile* growth (MIC ≈ 9.4 mg/mL) and even showed similar inhibiting potential to that obtained by vancomycin treatment (30 µg/disc; control) (≈30 mm inhibition zone, tested by means of Kirby–Bauer diffusion disc methodology). Moreover, aloe vera (14–19 mm inhibition zone) and artichoke products (12.7–13.9 mm inhibition zone) showed high antimicrobial potential against *C. difficile*. With regard to processed products (with dimethyl sulfoxide (DMSO) 20% primary solvent used in the extraction process), trans-cynnamaldehide (0.02% *v/v*), peppermint oil (8% *v/v*), coconut oil (32% *v/v*), allicin (MIC = 4.7 mg/mL; MBC = 37.5 mg/mL), and menthol (MIC = 9.4 mg/mL; MBC = 18.8 mg/mL) showed the most effective potential with the lowest required dosage (99.9% reduction of bacterial counts in microdilution test). The study by Roshan et al. [24] also revealed the synergistic effect between the natural compounds that were studied and conventional antibiotic therapies that are currently applied (vancomycin and metronidazole) (trans-cinnamaldehyde with metronidazole and trans-cinnamaldehyde with vancomycin), thereby opening new avenues for future treatment [24]. Moreover, hypervirulent (BI/NAP1/027) *C. difficile* strains and clinical toxigenic isolates showed susceptibility to curcuminoids, the major phytoconstituents of turmeric, at concentrations ranging from 4 to 32 μg/mL [23]. Curcumin was more effective than fidaxomicin in inhibiting *C. difficile* toxin production, with no negative effect on beneficial gut microbiota. Possible synergistic effects between curcumin and the most effective antibiotic therapies against *C. difficile* were also evaluated in vitro. Fidaxomicin (ranging from 0.0005 to 0.5 μg/mL), vancomycin, and metronidazole (at a range of 0.015–8 μg/mL) were tested in combination with curcumin (at a concentration range of 2–64 μg/mL). Although, no synergistic effect was detected between curcumin and the studied antibiotics, antagonist effects also did not manifest [23,24].

The studies by Aljarallah [38] and Num and Useh [26] also revealed the potential of natural herbal extracts to ameliorate possible CDI. In fact, bioactive molecules in herbal extract from *Nigella sativa* L. (including thymoquinone TQ) and *Myrrh* (*Commiphora myrrha*) showed a broad spectrum of antibacterial and antifungal activity against *C. difficile* (strains JIR and VPI) [26]. Both black seed oil (2% *v/v*) and *Myrrh* water extract (2% *v/v*) were effective in inhibiting growth of *C. difficile* in vitro (via the in vitro agar diffusion method) [23]. These results were consistent with those previously obtained by different researchers in relation to the effectiveness of these natural extracts against other gastrointestinal habitual pathogens, such as *E. coli, S. aureus, P. aeruginosa*, *Salmonella Typhimurium*, *S. flexneri, Bacillus circulans*, *Enterococcus faecalis*, and *Helicobacter pylori* [39,40]. 

A recent study by Yu et al. [25] demonstrated the effectiveness of manuka honey against 20 *C. difficile* clinical isolates, with MIC values for aqueous extracts in the range of 4 to >30% (*w/v*). Manuka honey (produced by *Apis mellifera* foraging *Leptospermum scoparium* flowers) demonstrated both bacteriostatic and bactericidal effects against this pathogen. It not only worked against planktonic cells but also inhibited *C. difficile* biofilm-forming capacity. Manuka honey also demonstrated optimum activity at 40–50% (*v/v*) concentration in inhibiting biofilm formation in four *C. difficile* ribotypes studied, namely R017, R023, R027, and R046 [41]. Manuka honey has been described as a nonallergenic product that does not have a negative impact on the gastrointestinal tract (GIT) microbiome. Its administration is also associated with stimulation of the epithelial cells and fibroblasts in the human host (increased resistance, thus preventing CDI) [27]. Additionally, manuka honey has been shown to be a more potent antimicrobial agent against Gram-positive than Gram-negative bacteria, which may be beneficial as adjunct therapy against CDI (preserving normal gut flora, which is predominantly Gram-negative) [41]. 

Natural essential oils (EOs) with well-recognized antimicrobial potential have also demonstrated an effective capacity for *Clostridium* spp. inhibition (*C. butyricum*, *C. intestinale*, *C. hystoliticum*, *C. perfringens*, and *C. ramosum*), but it has not yet been tested against *C. difficile* [25]. Among the studied EOs, *Satureia montana, Abies alba* Mill., and *Thymus vulgaris* were especially effective, with the lowest minimum inhibitory concentrations against *Clostridium* spp. (0.38–76 µL/mL) [27]. Mechanisms of action of natural antimicrobials in reducing bacterial cell viability include (i) effect on pH homeostasis and equilibrium of inorganic ions, (ii) inhibition of NADH oxidation, and (iii) structural and functional damage of the cell membrane. The cell wall of Gram-positive bacteria is constituted by a thick layer of peptidoglycan, contrary to Gram-negative bacteria that have a cell wall composed of a thin layer of peptidoglycan surrounded by an outer membrane (that is rich in lipopolysaccharides, in addition to proteins and phospholipids) [24]. The outer membrane of Gram-negative bacteria is often hidden by a slime layer, which in turn hides the antigens of the cell. This different structure (outer membrane of Gram-negative bacteria) prevents certain drugs and antibiotics from entering the cell, which means these bacteria have increased resistance to drugs. According to a recent study by Roshan et al. [24], one of the main advantages of these natural antimicrobials is that the antibiotic resistance mechanisms developed by *C. difficile* are not cross-protective for natural products. Furthermore, among the advantages derived from the application of naturally extracted antimicrobials is the minimal effect on gut microbiota by these treatments (*Bifidobacterium* spp., *Lactobacillus* spp., and *Bacterioides* spp. are less affected compared to conventional antibiotic strategies). This aspect is crucial in CDI progression and recurrence. Several studies have described dysbiosis in the GIT microbiome as a determinant factor in *C. difficile* colonization and subsequent infection [42,43,44]. Infected patients with *C. difficile* showed lower richness and diversity of beneficial gut bacteria (*Lactobacillus* spp. and *Bifidobacterium* *genera*) and also relative reduced abundance of *Bacteroidetes*, *Ruminococcaceae*, and *Lachnospiraceae* members [42,43,44]. A specific example was demonstrated by Crobach et al. [43] between control (noninfected CD individuals) and CDI patients. According to Crobach and co-workers, the presence of *Eubacterium hallii* and *Fusicatenibacter* contributed to generating resistance against *C. difficile* colonization and infection. In contrast, *Veillonella* is a genus that is always present in infected patients and related with susceptibility to CDI [43].

### 2.2. Probiotic Administration, Microbiota Restoration (Fecal Transplantation), and Microbiota Diet Modulation: A Biological Strategy to Improve CDI Resistance

Among the main disadvantages associated with antibiotic therapies in CDI management is the negative effect on normal microbiome of the host, which reduces a wide spectrum of protective microbiota (short-chain fatty acids (SCFA) producers and carbohydrate degraders such as *Eubacterium Hallii*, *Fusicatenibacter*, several *Enterococci*, *Ruminococcus gnavus*, and *Lachnoclostridium*) at the gastrointestinal level. It also reduces complete absorption of antibiotic from the intestinal tract, thereby restricting its concentration in the colon [36]. The International Human Microbiome Consortium and the National Institute of Health’s Human Microbiome Project (HMP) are undertaking research to explain how microbiome could play a critical role in human health and disease. Bacteroidetes (defined as groups able to break down host glycans and nondigestible carbohydrates, specifically resistant starches and plant cell wall polysaccharides) and Firmicutes (which form 50–70% of the colonic bacterial community), especially members of the *Clostridium* genus, are known for their ability to degrade polysaccharides and ferment amino acids (members of the Lachnospiraceae and Ruminococcaceae families) and have been described as always being present and predominant in healthy individuals. As CDI progresses, Proteobacteria and Bacteroidetes decrease [45]. 

Biological strategies such as microbiota transplantation and probiotic administration have also emerged as being effective in reducing and mitigating *C. difficile* infection [46]. Transplantation of fecal healthy microbiota (TFM) is currently being studied but is not yet a regulated strategy. According to in vivo studies carried out by Cammarota et al. [46] in CDI patients, TFM was effective in 90% of treated patients after just 1 year, with no adverse effects manifested. 

Regarding restoration of microbiota equilibrium in the gut, promising results have been obtained for biotherapeutic preparations of probiotics, which have been standardized and launched as nutraceuticals to combat recurrent *C. difficile* infections [46,47,48]. Examples of these preparations are RBX2660 and SER-109, which are in phase 3 (PUNCH CD (NCT03244644), 127 patients enrolled) and phase 2 (ECOSPORE, 87 patients enrolled) clinical trials, respectively. Rates of success close to 87% in CDI treatment were obtained using these biotherapeutic preparations, even in three times recurrent *Clostridioides* infection. No toxigenic effects were observed in any of the standardized microbiota mixtures, including purified Firmicutes spores (in the case of SER-109) [49,50]. At present, several companies are working on the development of similar biotherapeutic products (among them Pfizer, Nanotherapeutics, and Viropharma) to treat and reduce possible recurrent infection with *C. difficile*, such as the newly proposed product RBX7455 for oral *C. difficile* prevention, which is a first of its kind nonfrozen, room temperature stable oral microbiota-based formulation under the MRT™ drug platform. Most of them are at least in phase 2 clinical trials (2017–2020 period) and include both probiotic strategies to displace and mitigate CDI and vaccines specifically developed to prevent CDI [49,50]. 

Directly administered probiotics such as *Saccharomyces boulardii* l-745, *Lactobacillus rhamnosus* GG, *Lactobacillus plantarum* 299v, *Clostridium butyricum*, and *Lactobacillus acidophillus* have demonstrated good prospects in vitro in preventing *C. difficile* growth by means of an established competition between bacterial species in the media. However, to date, evidence from clinical trials regarding the potential benefits of probiotics against *C. difficile* is based exclusively on a few bacterial strains, meaning there is not enough data to generally accept and explain the positive in vivo potential [51,52,53,54]. In fact, to date, little is known about how the antagonism is established between probiotic bacteria and *C. difficile* proliferation [55,56]. The study by Khattab et al. [55] revealed *Lactobacillus* (*L. agilis*), *Enterococcus*, and *Clostridium* (mainly, *C. butyricum*) genera as having antagonistic potential against *C. difficile* by synthesis of extracellular thermostable antimicrobials. 

The beneficial effect of *S. boulardii* and *L. rhamnosus* GG has been further confirmed by the prevention of antibiotic-associated diarrhea, leading the ESPGHAN (European Society for Pediatric Gastroenterology Hepatology and Nutrition) to recommend the use of probiotics for the prevention of antibiotic-associated diarrhea in children [56]. Furthermore, the study by Chen et al. [57] revealed that genetically modified probiotic *S. boulardii* was able to constitutively secrete a single tetra-specific antibody that potently and broadly neutralized toxins secreted by *C. difficile* (TcdA and TcdB), demonstrating protection against primary and recurrent CDI in both prophylactic and therapeutic mouse models of disease [57]. 

Modulation of beneficial gastrointestinal bacteria by diet has also been described as a critical aspect contributing to preventing CDI. Jochems et al. [58] evaluated 18 dietary proteins (from protein sources whey, pea, egg, soyabean, insect, potato, fungi, corn, and yeast) to test the impact on epithelial cell colonization and toxin (TdcA and TdcB) production by *C. difficile*. According to the authors, diet supplementation with certain proteins can enhance the mitigating potential of host immune system to react and restore faster when CDI occurs. Egg-white protein increased IL-6 and IL-8 release (beneficial immunomodulatory effect of protein supplementation but preventing TcdA-induced disruptive consequences), while wheat, lesser mealworm, and yeast proteins increased nitric oxide levels after TcdA exposure. In the same research line, the study by Mefferd et al. [22] supported these previous conclusions. In addition to the specific effect of proteins in immune system reinforcement, Mefferd et al. demonstrated that carbohydrate-based diets exerted a protective effect against *C. difficile* gut colonization; in contrast, high fat/high protein diets, such as the Atkins diet, greatly exacerbated antibiotic-induced CDI. Hryckowian et al. [59] also found that mixtures of microbiota-accessible carbohydrates (MACs), specifically inulin, decreased *C. difficile* in vivo (humanized mice) by growth stimulation of carbohydrate-utilizing bacteria and SCFA production. The influence of carbohydrate-based diet on CDI prevention was also recently studied by Schnizlein et al. [44]. Xanthan gum (5% administered in the diet) was evaluated in vivo (C57BL/6 mice model) in terms of microbiota impact (16S rRNA gene amplicon sequencing). According to the results obtained in mice, the administration of xanthan gum increased fiber-degrading taxa and SCFA concentrations, altering mice susceptibility to *C. difficile* colonization (maintaining balanced microbiota). 

Modulation of gut microbial shape to reduce the ability of *C. difficile* to colonize and establish is among the most promising initiatives to prevent infection. For this task, diet can play a significant role as it can reduce *C. difficile* pathogenicity by not only regulating the ecological–microbial interactions in the gut but also altering the expression of pathogenesis factors. 

### 2.3. Marine Natural Compounds as Antimicrobials: Future Niche Strategy against C. difficile

In recent years (2010–2020), marine organisms have been increasingly considered as sustainable sources of food and pharmaceutical potential bioactives [60,61,62,63]. Bacteria, fish, shellfish, seaweed, microalgae, mollusks, crustaceans, and cephalopods, among others, are some of the biological matrices that have been identified as being able to produce or synthesize high added value metabolites with potential health benefits for humans [64,65,66]. Proteins, peptides, vitamins, carbohydrates, polyphenols, and terpenes are examples of marine molecules with demonstrated functional effects when accurately extracted, purified, and administered as food ingredients or pharmaceutical carriers. Prebiotic, antimicrobial, antioxidant, immunomodulatory, anticancerigen, lipidolemic, and angiotensin I-converting enzyme (ACE) activities are among the most relevant health benefits that have been exerted to date in vitro and in vivo by some of these molecules [64,65,66].

Algae marine organisms offer higher productivity rates than terrestrial plants (close to 12,000 dry tons of microalgal biomass is produced worldwide; protein efficiency/area unit macroalgae = 2.5–7.5 tn/ha/year; microalgae: 4–1 tn/ha/year) and can be sustainably produced as a source of valuable bioactives. The increasing pharmaceutical application of marine algae bioactives is mainly based on their demonstrated antioxidant, antimicrobial, and anticancerigen properties. Moreover, food and nutraceutical supplements based on raw or purified algae compounds are being developed [67,68,69]. 

Algae compounds have shown antibacterial potential against a wide range of Gram-positive and Gram-negative microorganisms [36,69]. The antimicrobial potential of algae materials is based on the (i) type and algae matrix source (e.g., different algae taxonomic groups, culture conditions, seasonal harvest, and accumulation of bioactives), (ii) structural chemical diversity of compounds, (iii) molecular weight of compounds, (iv) type of extraction and purification methods employed, and (v) modification and way of administration [23,54]. Among the most relevant antimicrobial bioactives from macro- and microalgae are phlorotannins, laminarin, sargafuran, peyssonoic acid, bromophycolides, neurymenolides, acetylmajapolene, phycobiliproteins, scytonemines, carotenoids, polysaccharides, phytohormones, cyanotoxins, phytol, fucosterol, neophytadiene, palmitic, palmitoleic, and oleic acids [36,37,63,67,69]. 

Seaweeds are classified into green algae, red algae, and brown algae based on their pigmentation. The most promising antimicrobial potential has been found in brown algae, namely Phaeophyceae (84% of species with demonstrated antimicrobial capability), followed by Rhodophyceae (67%) and Chlorophyceae (44%) [62]. Regarding microalgae, *Spirulina platensis* and *Chlorella vulgaris* are nowadays the most studied algae substrates in terms of their antibacterial/antiviral capacity [67,68,69,70].

Among the studied bacteria, some of the Gram-positive bacteria that are sensitive to algae compounds are strains of *Bacillus subtilis*, *Bacillus cereus*, *Staphylococcus aureus*, *Enterococcus faecalis*, and *Micrococcus luteus*, while the Gram-negative bacteria include *Klebsiella pneumoniae*, *Serratia marcescens*, *Escherichia coli*, *Pseudomonas aeruginosa*, *Salmonella* Typhimurium, and *Vibrio cholerae*. Clinical human pathogens, such as *P. aeruginosa*, *E. coli*, *S. aureus*, *E. faecalis*, group B *Streptococcus* (GBS), and *Proteus mirabilis*, are among pathogens that most frequently affect hospitalized patients, and all of them have demonstrated sensitivity to exposure to natural compounds from marine algae sources [36,37,69,70,71,72,73,74]. Five microalgae cultures (*Chlorella minutissima, Tetraselmis chui, Nannochloropsis* sp., *Arthrospira platensis*, and *Isochrysis* sp.) were effective in inhibiting Gram-positive and Gram-negative nosocomial pathogens, with MIC value equal to 300 µg/mL for *Chlorella vulgaris* and *Spirulina platensis* against the most resistant clinical pathogens under study [28]. The chemical characterization of algae extracts demonstrated that volatile algae oils contained in *Chlorella* spp. and *Spirulina platensis*, including linalool, geraniol, citronellol, monocyclic limonene, 1-8-cineol, p-cymene, bicyclic α-and β-pinene, cadinene, aromatic eugenol, and isoeugenol, exerted a potent antimicrobial effect against the studied bacteria [28]. Moreover, terpenes (such as π-cymene (+), limonene, β-myrcene, β-pinene, and linalool) have shown active antimicrobial potential toward drug-resistant pathogens [28,29].

To our knowledge, in spite of the extensive existing literature related to the assessment of natural algae antimicrobial bioactives against a wide spectrum of human/animal pathogens, nothing has been previously reported in relation to the effectiveness of algae compounds in inhibiting *C. difficile* proliferation. We can, however, consider that algae compounds with demonstrated prebiotic potential (mainly polysaccharides and complex sulfated bioactives) in promoting significant improvement of healthy microbiota could consequently also improve resistance of the GIT microbial population against CD colonization and reinforcement of the host immune system, thereby preventing several infection recurrence episodes. Similar approaches have been developed to study and prevent one of the most concerning gastrointestinal pathogens, the unique biological carcinogenic agent *Helicobacter pylori* [66,75]. 

Algae compounds with recognized antimicrobial and protective digestive effects as well as matrices rich in algae polysaccharides matrices have been described as being useful as prebiotics. *Laminaria, Saccharina, Spirulina platensis, Chlorella* species, *Dunaliella salina*, and *Scenedesmus* species have been shown to exert a potent prebiotic capability by oral administration and also by integration in the diet of in vivo animal models [28,29,76]. Several studies focusing on algae polysaccharides have recently been published dealing with the positive impact of diet rich in algae polysaccharides on human gut microbiota balance and its possible capacity to reinforce the host response against *C. difficile* invasion. According to the study by Han et al. [30], the abundance of Ruminococcaceae, Coprococcus, Roseburia, and Faecalibacterium in an animal model was increased by diet supplementation of polysaccharides and oligosaccharides. Meanwhile, diet supplementation of algae polysaccharides and oligosaccharides has been shown to have a positive impact in preventing proliferation of opportunistic pathogenic bacteria *Escherichia, Shigella*, and *Peptoniphilus*. Among macroalgae polysaccharides, special attention has been paid in the last decade to fucoidan (sulfated polysaccharide rich in fucose) from brown macroalgae (Phaeophyceae). The antimicrobial potential of fucoidan has been recognized in several high-impact studies against gastrointestinal pathogens [31,36,37,77]. Purified fucoidan showed effective MIC concentrations in the range of 25–100 µg/mL against *Salmonella enterica* serovar Typhimurium and *Helicobacter pylori* depending on the algae source (*Fucus vesiculosus, Undaria pinnatifida*, and *Macrocystis pyrifera*) [31,66]. Fucoidan from *Fucus vesiculosus* was most effective against the studied gastrointestinal pathogens. Since 2017, fucoidan from *Fucus vesiculosus* and *Undaria pinnatifida* have been granted “Generally Recognized as Safe” (GRAS) designation by the US FDA and received EU Novel Foods approval. Another study on supplementation of fucoidan to human diet revealed how fecal innate immunity indicators were improved (e.g., lysozyme concentrations, expression of key intestinal tight junction proteins, and secretion of antimicrobial peptides in the gut mucosa) [77]. 

Moreover, polysaccharide chitosan (natural cationic polysaccharide composed of randomly repeating units of β-(1,4)-linked D-glucosamine (deacetylated unit) and N-acetyl-D-glucosamine (acetylated unit) nanofibers with extended application in food formulation and packaging were recently (2020) described as having antimicrobial properties against clinical toxigenic isolates of *C. difficile* with promising in vitro results (MIC values of 0.25 µg/mL) [32]. 

Phocoenamicin, a novel natural compounds from marine mammal microbiota (from *Micromonospora auratinigra*, Actinobacteria), has recently been extracted, purified, and characterized with a potent selective antimicrobial activity against *C. difficile* [78,79]. In fact, marine ecological habitats have huge microbial diversity, with high capability to synthesize antimicrobial substances. Among these natural sources of antimicrobials, Actinobacteria (from marine sediment habitat) have been recognized as major producers of antimicrobial compounds [34]. Actinobacteria (*Actinomadura, Actinoplanes, Amycolatopsis, Marinispora, Micromonospora, Nocardiopsis, Saccharopolyspora, Salinispora, Streptomyces*, and *Verrucosispora*), as prolific producers of pharmaceutical metabolites (70% of bioactives produced by Actinobacteria are currently in clinical use), are producing potent antimicrobials that could be applied in nutraceuticals for future prevention of CDI. Thiocoraline (peptide) from *Micromonospora* sp., bonactin (esters) from *Streptomyces* sp., and chinikomycins A from *Streptomyces* sp. have been shown to exert potent antimicrobial activity against Gram-positive bacteria (MIC ≈ 4 µg/mL) [35]. In the period, 2015–2018, 45 patents have been launched claiming therapeutically active biomolecules from marine sources, mainly aimed at treating or preventing cancer, infectious diseases, and cardiovascular disorders [79]. Among these novel products with unique structures and novel bioactivity are, isoquinoline alkaloid; trabectedin, the polyether macrolide; halichondrin B, and peptide dolastatin 10 [34,35,79].

Recently presented results on marine substrates are opening new possibilities in terms of CDI treatment by means of different strategies, namely (i) pharmaceutical products designed to be applied to complement antibiotic therapies against *C. difficile* and (ii) possible diet supplementation with these marine prebiotics and highly nutritional molecules (e.g., peptides) that may exert an additional antimicrobial effect against *C. difficile* gut invasion.

## 3. Conclusions

Novel developments in the field of antimicrobial therapies against *C. difficile* are now under way. The urgent need to find effective antimicrobial strategies to fight against this pathogen without affecting beneficial microbiota at the GIT level is one of the main challenges to achieve highly specific treatment. Tailor-made antimicrobial strategies should be developed against CDI for both (i) prevention and (ii) treatment (depending on the severity of symptoms manifested and previous clinical history of the patient). Natural compounds from vegetable and marine origin are being investigated due to their anticlostridial bacteriostatic and bactericidal potential as well as their capacity to maintain healthy microbiota equilibrium. Special attention should be paid to algae compounds as sustainable and worthy sources of unexplored antimicrobials. Fucoidan from *Phaeophyceae* is among these valuable compounds with demonstrated prebiotic potential. It promotes the proliferation of beneficial bacteria while exerting antimicrobial effect against gastrointestinal pathogens such as *Helicobacter pylori* and *Salmonella enterica*. Further research is required on the use of algae antimicrobials as nutraceuticals in CDI management. Structurally effective, sustainable, easily extracted, and cost-effective purified natural biomolecules will be a reality in the short to medium term based on these antimicrobial compounds from vegetable and algae origins, which can be used an alternative to antibiotic-based therapy (diet or nutraceutical administration of natural compounds alone) or as a supplement to drugs (with possible synergistic effects) against *C. difficile*. In vivo studies to further understand (i) to what extent these compounds are available and effective in the digestive tract to exert antimicrobial functionality and (ii) how long nutraceuticals should be administered to ensure a protective effect on *C. difficile* colonization are required for safe and effective risk mitigation against CDI. 

## Figures and Tables

**Table 1:** Antimicrobial therapies against *Clostridioides difficile*: prospective drugs and natural compounds for CDI management.

Antibiotics and Natural Antimicrobials	Concentration	*C. difficile* (Inhibition mm)	References
Antibiotics	MIC (µg/mL)/MBC (µg/mL)		
Vancomycin (VAN)	0.5–4	-	[2,4]
Metronidazole (MTZ)	0.25–16	-	[2,4]
Fidaxomicin (FDX)	0.015–1	-	[9,23]
Ibezapolstat	2–4	-	[3]
Cadazolid	0.12–0.25	-	[12]
Vegetable Origin Matrices	MIC		
Vancomycin (positive control)		30.3 ± 0.7	[24]
Onion juice	100% (*v/v*)	10.3 ± 0.6	
Garlic juice	100% (*v/v*)	27.0 ± 1.0	
Ginger juice	100% (*v/v*)	-	
Garlic powder	(20% *w/v*)	26.6 ± 0.6	
Cinnamon powder	(20% *w/v*)	20.9 ± 0.9	
Curcumin	4–32 µg/mL	md	[25]
Manuka honey	50% (*v/v*)	11.4 ± 0.5	[26]
*Nigella sativa* (black seed oil)	2% (*v/v*)	>15	[27]
*Commiphora myrrha* (water extract)	2% (*v/v*)	>15	
EOs (*Satureia montana, Abies alba* Mill., and *Thymus vulgaris*)	50 µL/mL	>20	[28]
Marine Antimicrobials			
*Chlorella* spp. and *Spirulina platensis* EOs	300 µg/mL	8–21	[29,30]
Polysaccharides from *Laminaria, Saccharina, Spirulina platensis, Chlorella* species, *Dunaliella salina,* and *Scenedesmus*	25–100 µg/mL	md	[31,32]
Chitosan	0.25 mg/mL	md	[33]
Phocoenamicin (from *Micromonospora auratinigra*)	2.6 µM	>15	[34]
Thiocoraline (peptide) from *Micromonospora* sp.; bonactin (esters) and chinikomycins A from *Streptomyces* sp.	4 µg/mL	>15	[35]

MIC: minimum inhibitory concentration; EOs: essential oils; md: growth inhibition assayed by microdilution method.

## Data Availability

Data sharing is not applicable to this article.
